# Host Pre-Adaptation and Sublethal Indoxacarb Interactively Affect Growth and Development of *Cnaphalocrocis medinalis*, with Potential Involvement of P450 and Juvenile Hormone-Related Genes

**DOI:** 10.3390/insects17101043

**Published:** 2026-10-10

**Authors:** Sunan Shen, Ronggen Wen, Yajun Yang, Hongxing Xu, Yu Chen, Can Li, Yanhui Lu, Yan Liu, Yuxuan Chen, Zhongxian Lu, Yan Wu, Jiawen Guo

**Affiliations:** 1 Key Laboratory of Surveillance and Management of Invasive Alien Species, Guizhou Key Laboratory of Agricultural Biosecurity, Department of Biology and Engineering of Environment, Guiyang University, Guiyang 550005, China; 2 State Key Laboratory for Quality and Safety of Agro-Products, Institute of Plant Protection and Microbiology, Zhejiang Academy of Agricultural Sciences, Hangzhou 310021, China; 3 Shengzhou Agricultural Technology Extension Center, Shaoxing 312400, China

**Keywords:** *Cnaphalocrocis medinalis*, host switching, indoxacarb, sublethal effect, P450, juvenile hormone

## Abstract

Host switching alters insecticide responses, but its interplay with sublethal stress remains unclear. We examined if host pre-adaptation modulates sublethal indoxacarb effects on *Cnaphalocrocis medinalis* and whether P450 and juvenile hormone-related genes are involved. Two long-term laboratory populations with different origins and rearing histories were used: a wheat-reared population (collected 2019 in Nanjing; >20 generations) and a rice-reared population (collected 2021 in Hangzhou; >10 generations). They were reciprocally transferred onto rice and wheat, establishing four host switching treatments (RR, RW, WR, WW). Second-instar larvae were exposed to LC_25_ indoxacarb, after which we recorded larval and pupal development, fecundity and egg hatchability, and quantified six P450 genes (*CmedCYP49A1*, *CmedCYP6AE30*, *CmedCYP12A2*, *CmedCYP301A1*, *CmedCYP4V2*, and *CmedCYP4AE76*) and four JH-related genes (*CmedAT*, *CmedJHAMT*, *CmedJHE*, and *CmedJHBP1*). Host switching was associated with altered susceptibility, with RR more susceptible than RW and WW less susceptible than WR. Developmental effects were instar-specific. Pupal traits were affected by insecticide treatment and population origin, whereas reproductive traits were mainly affected by population origin. P450 and JH-related genes showed gene-specific expression patterns and were associated with some phenotypic changes. Gene expression correlated with phenotypes, suggesting potential trade-offs among detoxification, development, and reproduction. These laboratory findings indicate that host pre-adaptation and sublethal indoxacarb affect life-history traits in trait- and instar-specific ways. P450 and JH genes are potentially involved and merit functional validation, but field validation is needed before management application.

## Simple Summary

Migratory pests move between fields and encounter different host plants, which can alter their responses to insecticides. However, it is unclear whether the host plant a pest previously fed on affects how low-dose insecticides influence its growth and reproduction. In this study, we used rice and wheat to create four host switching treatments in *Cnaphalocrocis medinalis*, an important migratory rice pest. We exposed larvae to a low dose of indoxacarb and measured their development, survival, reproduction, and the expression of genes involved in detoxification (P450 genes) and hormone regulation (juvenile hormone-related genes). We found that host plant history significantly changed larval sensitivity to indoxacarb. The effects of indoxacarb on development were instar-specific: it prolonged development in most treatments but shortened it in the WR treatment. P450 and juvenile hormone-related genes showed different expression patterns depending on host background, and these changes were associated with differences in growth and reproduction. Our findings suggest that previous host plant experience may be associated with altered insecticide responses, with P450 and juvenile hormone genes potentially involved. Understanding this relationship may help generate hypotheses for future studies on insecticide response and susceptibility monitoring, but further functional and field validation is required before practical or resistance-management recommendations can be made.

## 1. Introduction

The utilization of host plants by herbivorous insects is not a static process, but a dynamic adaptive outcome shaped by ongoing plant-insect interactions. For polyphagous pests or migratory pests with broad host ranges, host plant switching triggers multi-level physiological plasticity, including changes in nutrient utilization, detoxification metabolism, gut microbial composition, and growth and development [1,2]. Recent studies have shown that host-induced adaptation can not only affect how insects utilize plants, but also alter their responses to external compounds. For example, *Spodoptera frugiperda* shows different development, detoxification gene expression, gut microbiota, and insecticide tolerance when reared on maize versus rice [3]. *Grapholita molesta* exhibits changes in P450 expression profiles, gut microbes, and susceptibility to emamectin benzoate after switching feeding sites [4]. Plant secondary metabolites can activate the insect detoxification system and lead to pre-adaptation to insecticides. For instance, the maize defense compound DIMBOA (2,4-dihydroxy-7-methoxy-1,4-benzoxazin-3-one) induces the detoxification system in *Spodoptera litura*, increasing the activity of detoxification enzymes such as cytochrome P450 monooxygenase (P450), carboxylesterase (CarE), and glutathione S-transferase (GST), and upregulating the expression of detoxification-related genes like P450 and UDP-glycosyltransferase (UGT), making the insect more tolerant to both plant toxins and pesticides [5]. The Akt-FoxO signaling pathway can coordinate the expression of multiple P450 genes, allowing herbivorous insects such as *Nilaparvata lugens* to co-adapt to plant defensive secondary compounds and insecticide stress [6]. These studies suggest that there is likely a shared or linked metabolic pathway between host plant pre-adaptation and chemical insecticide stress, and such a link may ultimately interactively affect insect growth, development, and reproduction. Although sublethal effects have been shown to harm insects in multiple ways [7,8], it remains unclear whether there is a significant interaction between host plant pre-adaptation and sublethal insecticide effects, and how such an interaction influences pest growth, development, and reproduction. Therefore, gaining a deeper understanding of this interaction mechanism is of great theoretical and practical importance for revealing the adaptive evolution mechanisms of pests and optimizing integrated pest management strategies in the field.

As a key detoxification system for insects responding to insecticide and plant defense stresses, the cytochrome P450 monooxygenase system plays a central role in host-induced cross-adaptation [9,10]. In *S. frugiperda*, silencing *CYP321A7* and *CYP6AE43* increases larval susceptibility to indoxacarb [11]. In *S. litura*, multiple overexpressed P450s have been confirmed to participate in the metabolic conversion of indoxacarb [12]. Among the P450 enzyme family, *CYP6AE30* and *CYP4AE76* are closely related to xenobiotic metabolism in lepidopteran insects [10,13]. Apart from detoxification pathways, juvenile hormone (JH) signaling is a core endocrine axis regulating insect growth, development, molting, metamorphosis, and reproduction [14]. Sublethal doses of insecticides can also interfere with the JH pathway. For example, indoxacarb treatment significantly reduces the JH III titer in larvae of *Chilo suppressalis* and upregulates the expression of the key degradation enzyme juvenile hormone esterase (JHE) [15]. In *Nilaparvata lugens*, sublethal emamectin benzoate promotes JH synthesis and increases fecundity [16]. JH homeostasis is finely regulated by genes involved in its synthesis (allatotropin, AT; juvenile hormone acid methyltransferase, JHAMT), degradation (JHE), and binding and transport (juvenile hormone binding protein, JHBP) [16,17]. Notably, the P450 system and the JH pathway are not independent of each other. Studies have shown that, while detoxifying xenobiotics, P450s are also key enzymes in the JH biosynthetic pathway. For instance, the CYP15 family catalyzes the terminal epoxidation step of JH synthesis [18,19], and some P450 genes even possess dual functions in endogenous hormone synthesis and xenobiotic metabolism [20]. Research has found that CYP337B5 not only participates in the metabolism of chlorantraniliprole but is also specifically involved in the synthesis of JH I and JH II [21]; the CncC signaling pathway mediates the trade-off between detoxification and reproduction by simultaneously regulating the expression of P450 detoxification genes and JH degradation enzyme genes [20]. This indicates that P450s are involved in the JH regulatory network. Therefore, it is speculated that host plant pre-adaptation and sublethal insecticide stress may interact through the coordinated regulation of the P450 detoxification network and the juvenile hormone signaling axis.

*Cnaphalocrocis medinalis* is one of the most important pests in Asian rice-growing regions [22]. As a migratory insect, *C. medinalis* flies long distances in Southeast Asia several times a year and usually migrates downwind and lands randomly [23], implying that it may encounter different host plants after migration. Larvae of *C. medinalis* can infest both rice and wheat [24,25]. Although rice and wheat are both cereal crops, they differ in their defensive chemistry and nutritional quality [26,27]. Long-distance migration may cause *C. medinalis* to switch between rice and wheat in the field, and such host switching is ecologically relevant because it can alter larval fitness, detoxification enzyme activities, and susceptibility to insecticides [1]. Chemical control remains the primary management approach, although long-term insecticide use has led to increasing resistance problems [22,28]. Currently, indoxacarb, a voltage-dependent sodium channel blocker [29], is one of the few highly effective insecticides to which *C. medinalis* still maintains relatively high susceptibility, making it valuable for insecticide rotation and resistance management [28]. Previous studies have shown that host switching can affect the susceptibility of *C. medinalis* to abamectin and chlorpyrifos [30], demonstrating that host adaptation may alter pest responses to insecticides. However, how host pre-adaptation influences the sublethal effects of indoxacarb in *C. medinalis*, and the underlying molecular mechanisms, remain unclear. It is established that P450 detoxification enzymes and the juvenile hormone signaling pathway are two key systems mediating insect responses to xenobiotic stress, and that they are closely interconnected [9,20]. Based on this, we propose the hypothesis that host pre-adaptation and sublethal indoxacarb may interactively affect the growth and development of *C. medinalis*, and that this interaction may be associated with changes in the expression of P450- and juvenile hormone-related genes.

To test the above hypotheses, we used second-instar larvae derived from host-switching between two populations (a rice-reared and a wheat-reared population) as experimental materials. We first determined the susceptibility of larvae with different host origins to indoxacarb, then systematically evaluated the combined effects of host switching and sublethal indoxacarb treatment on larval development, pupal traits, and adult reproduction. We also examined the expression changes in selected P450 genes (*CmedCYP49A1*, *CmedCYP6AE30*, *CmedCYP12A2*, *CmedCYP301A1*, *CmedCYP4V2*, and *CmedCYP4AE76*) and juvenile hormone-related genes (*CmedAT*, *CmedJHAMT*, *CmedJHE*, and *CmedJHBP1*), and analyzed the correlations between molecular responses and phenotypic variations. This study will provide experimental evidence for understanding the interactive effects of host pre-adaptation and sublethal insecticide stress, and offer a reference for the rational use of indoxacarb and resistance management in *C. medinalis*.

## 2. Materials and Methods

### 2.1. Insect Rearing

Two host-associated laboratory populations of *C. medinalis*, a wheat-reared population and a rice-reared population, were used in this study. To minimize the influence of environmental differences on insect status, all insects were maintained in climate-controlled chambers. Unless otherwise specified, the rearing and experimental conditions were standardized at 25 ± 1 °C, 80–90% relative humidity, and a photoperiod of 14 h light to 10 h dark.

The wheat-reared population was first collected from rice fields in Nanjing, Jiangsu Province, China (118.78° E, 32.06° N), in 2019 and subsequently maintained for more than 20 consecutive generations on wheat seedlings under controlled laboratory conditions [30]. The wheat cultivar used was Zhefeng 2. Wheat seeds were soaked in water for 24 h, pregerminated for 24 h, and then sown in disposable plastic cups with perforated bottoms for seedling cultivation. When the seedlings reached 8 to 10 cm in height, they were used for larval rearing. The rice-reared population was first collected from rice fields in Xiaoshan, Hangzhou, Zhejiang Province, China (120.22° E, 30.04° N), in 2021 and subsequently maintained for more than 10 consecutive generations on rice seedlings under controlled laboratory conditions. The rice cultivar used was TN1. Rice seeds were soaked in water for 24 h and pregerminated for 48 h, and sowing was conducted in the same manner as for wheat. Rice seedlings were used for larval rearing after 45 days of growth.

Both host plants were grown in the greenhouse of the Zhejiang Academy of Agricultural Sciences (Hangzhou, China). No pesticides were applied during cultivation, and the plants showed no obvious symptoms of diseases or insect damage. After mature larvae pupated, each pupa was placed individually in a ventilated plastic box lined with absorbent cotton until adult emergence. Newly emerged adults were transferred to 500-milliliter rearing cups and provided with cotton soaked in 5% honey solution as a supplementary food source; the cup opening was covered with plastic wrap to provide an oviposition substrate.

### 2.2. Host Switching Treatments

To establish different combinations of population origin and feeding host, eggs were collected from the wheat-reared and rice-reared populations and transferred onto wheat or rice seedlings. Four treatments were set up based on population origin and host plant: larvae of the rice-population reared on rice (RR), larvae of the rice-population reared on wheat (RW), larvae of the wheat-population reared on rice (WR), and larvae of the wheat-population reared on wheat (WW). All treatments were maintained in cages (50 cm × 50 cm × 50 cm). Fresh host plants were supplemented or replaced regularly throughout the experiment to ensure an adequate food supply for larvae. Larval growth and development were recorded daily, and individuals were sampled at the second instar for subsequent experiments.

### 2.3. Sublethal Concentration Determination of Indoxacarb and Bioassays of Cnaphalocrocis medinalis

To determine the susceptibility of *C. medinalis* to indoxacarb under different host switching conditions and to establish the sublethal concentration (LC_25_), bioassays were conducted using the four host switching treatment groups (RR, RW, WR, and WW). The formulated insecticide used in the bioassay was 99% indoxacarb (Shaanxi Huicheng Biotechnology Co., Ltd., Xi’an, China). A stock solution of 1000 mg/L was prepared by dissolving indoxacarb in acetone, and working solutions were generated by serial dilution with distilled water. Based on preliminary experiments, eight concentrations were tested: 0 (control), 0.003, 0.006, 0.0125, 0.025, 0.05, 0.1, and 0.2 mg/L, to determine the LC_25_. Bioassays were conducted using the leaf-dipping method: rice or wheat leaves (approximately 10 cm in length) were immersed in the insecticide solution for 30 s, air-dried, wrapped with moistened absorbent cotton at the base, and placed in Petri dishes lined with filter paper at the bottom. Fifteen second-instar larvae from each host switching treatment (starved for 2 h prior to the assay) were randomly selected and transferred to the Petri dishes containing treated leaves. Three replicates were performed for each concentration of indoxacarb. After 48 h of exposure, larval mortality was recorded. Solvent-only controls were included in all treatments. Each bioassay was performed with three biological replications.

### 2.4. Effects of Sublethal Indoxacarb on Growth, Development, and Reproduction of Cnaphalocrocis medinalis Under Different Host Switching Treatments

To evaluate the effects of LC_25_ indoxacarb on growth, development, and reproduction of *C. medinalis*, the four host switching treatments (RR, RW, WR, and WW) were established. Because the susceptibility of the four host-switching treatments to indoxacarb differed, each treatment was exposed to its own previously determined LC_25_ concentration, representing equal mortality pressure rather than equal absolute concentration. For each treatment combination, three independent biological replicates were set up. Each replicate consisted of one plastic box (20 cm × 10 cm × 10 cm) containing 200 s-instar larvae. Larvae in each treatment were exposed to the determined LC_25_ indoxacarb, with corresponding solvent-only controls for each treatment. After 48 h of exposure, surviving larvae were transferred to 12-cm Petri dishes lined with filter paper and supplied with fresh host leaves, with five larvae per dish, and reared until pupation. Each treatment group (each host-switching treatment under control or LC_25_ exposure) retained at least 30 surviving larvae for larval instar duration recording. The durations of the third-, fourth-, and fifth-instar larvae were recorded by daily examination. For each 12-cm Petri dish, the durations of the surviving larvae were averaged to obtain the mean duration of the third, fourth, and fifth instars per dish.

Because *C. medinalis* larvae roll leaves and feed inside leaf rolls, repeated opening of the leaf rolls for instar determination may cause mechanical damage. Therefore, the larvae used for measuring pupal weight, pupation rate, and adult reproduction were not subjected to larval instar examination. For each treatment group, at least 30 surviving larvae were retained for these measurements. These larvae were reared using the same individual rearing method described above, but without opening the leaf rolls for instar recording. When larvae stopped feeding, the leaf rolls were opened to collect prepupae. Pupation rate was calculated for each Petri dish as the number of pupated individuals divided by the total number of surviving larvae initially placed in that dish. After pupation, the filter paper was kept moist by daily addition of water to prevent desiccation. On the third day after pupation, live pupae were individually transferred to new Petri dishes and weighed; dead pupae were not weighed. Each new Petri dish was lined with moist filter paper. Because each pupa was then kept singly, each pupa in its own Petri dish was considered an independent biological replicate. After weighing, each pupa was kept individually in its Petri dish until adult emergence. After adult emergence, one newly emerged female and one newly emerged male from the same treatment were paired (1:1) and reared in a 350-mL plastic cup. The cup opening was covered with plastic wrap for oviposition, and a cotton ball soaked in 5% honey solution was placed at the bottom. The number of eggs laid per female was recorded. Egg sheets were placed in 12-cm Petri dishes lined with moist filter paper. Egg hatching was observed daily, and the filter paper was kept moist. Hatchability was calculated.

### 2.5. RNA Extraction, Reverse Transcription, and Quantitative Real-Time PCR

We previously conducted transcriptomic sequencing on *C. medinalis* populations subjected to the four host switching treatments (RR, RW, WR, and WW). These data are unpublished but have been deposited in the Genome Sequence Archive (GSA) at the National Genomics Data Center (NGDC), China (CRA026193, CRA026192, CRA026190, and CRA026182). Analysis of differentially expressed genes (DEGs) followed by KEGG annotation revealed that host switching induced 2 and 13 P450 DEGs in the rice and wheat populations, respectively, providing 14 P450 candidates in total. From these candidates, we selected six P450-related genes (*CmedCYP49A1*, *CmedCYP6AE30*, *CmedCYP12A2*, *CmedCYP301A1*, *CmedCYP4V2*, and *CmedCYP4AE76*) for expression analysis because they showed significant differential expression among host-switching treatments, had relatively high expression abundance, and belonged to P450 families previously associated with xenobiotic metabolism or insecticide detoxification in lepidopterans. In addition, we selected four key genes involved in juvenile hormone biosynthesis and metabolism pathways (*CmedAT*, *CmedJHAMT*, *CmedJHE*, and *CmedJHBP1*) to examine their expression responses after 48 h of LC_25_ indoxacarb exposure under different host switching backgrounds. Larvae used for gene expression analysis were randomly collected from the surviving larvae in the three plastic boxes (biological replicates) of each treatment group described in Section 2.4 after 48 h of LC_25_ indoxacarb exposure. For each treatment group, five surviving larvae were randomly selected from each of the three plastic boxes and pooled as one biological replicate, yielding three biological replicates per treatment group.

Total RNA extraction was performed using the TRIzol method following Zhao et al. [31]. Briefly, frozen larval samples were homogenized in TRIzol reagent (Tiosbio, Beijing, China), followed by chloroform extraction, isopropanol precipitation, and 75% ethanol washing. RNA was dissolved in DEPC-treated water. RNA integrity was examined by agarose gel electrophoresis, and concentration and purity were measured using a NanoDrop 2000 spectrophotometer (Thermo Fisher Scientific, Wilmington, DE, USA). First-strand cDNA was synthesized using the Polestar 1st cDNA Synthesis Kit (Tiosbio, Beijing, China) according to the manufacturer’s instructions. Quantitative real-time PCR was performed using iTaq™ Universal SYBR^®^ Green Supermix (Bio-Rad Laboratories, Hercules, CA, USA) on a Bio-Rad real-time PCR system, with *Cmedβ-actin* as the reference gene (primer sequences listed in Table 1). The amplification protocol was: 95 °C for 30 s, followed by 40 cycles of 95 °C for 5 s and 60 °C for 34 s, with a melt-curve analysis at 95 °C for 15 s, 60 °C for 60 s, and 95 °C for 15 s. Each biological sample was run in three technical replicates, and relative expression levels were calculated using the 2^−ΔΔCt^ method [32].

### 2.6. Statistical Analysis

Bioassay data for susceptibility were analyzed using POLO-Plus software (Version 2.0) to calculate the slope and its standard error, as well as the LC_25_ and LC_50_ values and their 95% confidence intervals [33]. The Shapiro–Wilk test was first used to assess the normality of data distributions for the four host switching treatment groups (RR, RW, WR, and WW) under both sublethal indoxacarb exposure and control conditions for all measured continuous variables, including larval developmental durations (third, fourth, and fifth instars), pupal weight, pupation rate, fecundity (number of eggs laid per female), egg hatchability, and the relative expression levels of P450- and JH-related genes, and Levene’s test was used to test homogeneity of variances for these parameters. Percentage data were subjected to arcsine square root transformation before analysis. Since the developmental durations of the third-, fourth-, and fifth-instar larvae, pupal weight, pupation rate, number of eggs laid per female, and egg hatching rate did not meet the assumptions of normal distribution, Scheirer–Ray–Hare tests was employed to examine the main and interactive effects of insecticide treatment (control vs. LC_25_), population origin (rice-reared vs. wheat-reared), and host switching (switched vs. non-switched) on these parameters, followed by Mann–Whitney U tests for pairwise comparisons between groups. Since the relative expression level data (2^−ΔΔCt^ values) of P450- and JH-related genes met the assumptions of normal distribution, three-way analysis of variance (ANOVA) was used to examine the effects of insecticide treatment, population origin, host switching, and their interaction, followed by *t*-tests for pairwise comparisons between groups. For Spearman correlation analysis, the three life-history replicate values per treatment group (each derived from one plastic box described in Section 2.4) were paired with the three gene expression biological replicates from the corresponding plastic boxes described in Section 2.4, to evaluate the relationships between the expression levels of P450- and JH-related genes and these key biological parameters. All data analyses and visualizations were performed using R (version 4.2.1).

## 3. Results

### 3.1. Susceptibility of Larvae to Indoxacarb from Different Host Switching Treatments

The susceptibility of *C. medinalis* larvae to indoxacarb varied among the four host switching treatments (Table 2). The RR treatment (rice-population reared on rice) exhibited the highest susceptibility, with the lowest LC_50_ value of 0.016 mg/L. In contrast, the WW treatment showed the lowest susceptibility, with an LC_50_ of 0.567 mg/L, corresponding to a 17.11-fold decrease in sensitivity compared with the RR group. The RW (rice-population reared on wheat) and WR treatments exhibited intermediate susceptibilities, with LC_50_ values of 0.115 mg/L (relative toxicity = 2.37) and 0.022 mg/L (relative toxicity = 4.89), respectively. The LC_25_ values followed a similar trend, ranging from 0.001 mg/L in the RR treatment to 0.064 mg/L in the WW treatment. These results suggest that host switching significantly affects larval susceptibility to indoxacarb, with the WW treatment showing the greatest reduction in sensitivity, while the WR treatment partially restored susceptibility compared with WW.

### 3.2. Sublethal Effects of Indoxacarb on Developmental Duration of Different Instar Larvae from Different Host Switching Treatments

The developmental duration of *C. medinalis* larvae at different instars was significantly affected by LC_25_ indoxacarb exposure, population origin, and host switching background, as well as their interaction, with effects varying across instars (Table 3).

**Figure 1:**
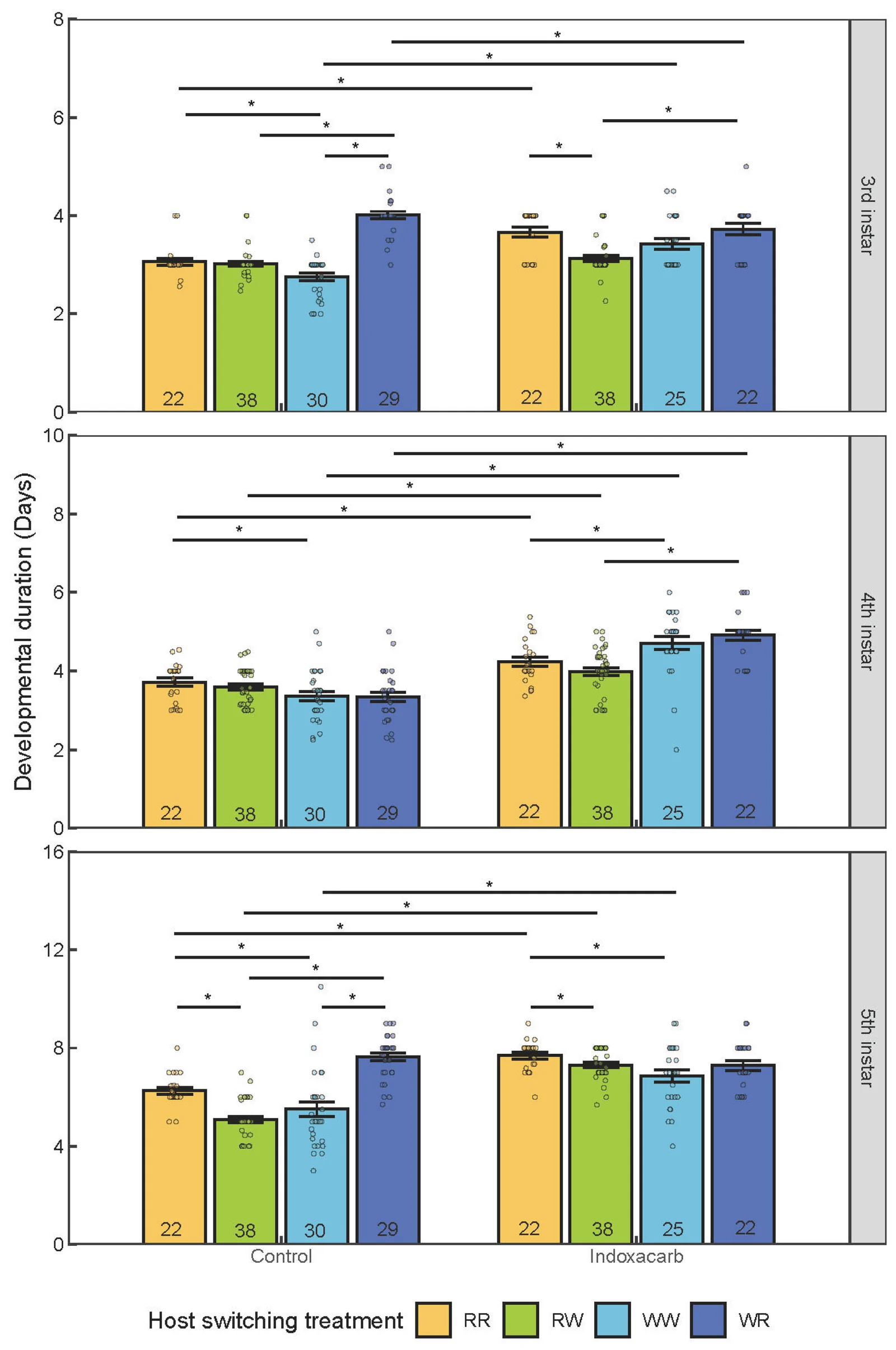
Developmental duration of *C. medinalis* larvae at different instars under LC_25_ indoxacarb exposure and different host switching backgrounds. Dots represent individual sample values. Bars represent means, and error bars represent standard errors (mean ± SE). The numbers on the bars indicate sample sizes. Asterisks indicate significant differences among different treatment groups (Mann–Whitney U test-*p* < 0.05). Host switching abbreviations: RR, rice-population reared on rice; RW, rice-population reared on wheat; WR, wheat-population reared on rice; WW, wheat-population reared on wheat.

For the 3rd instar, insecticide treatment, population origin, and host switching treatment all exerted significant effects, and the interactions between insecticide treatment and host switching treatment and between population origin and host switching treatment were also significant (Table 3). In the control groups, larvae in the RR treatment exhibited significantly longer developmental duration than those in the WW treatment (*W* = 439.5, *p* = 0.017) (Figure 1). In addition, larvae in the WW treatment exhibited significantly shorter developmental duration than those in the WR treatment (*W* = 857.5, *p* < 0.001), and larvae in the RW treatment also exhibited significantly shorter developmental duration than those in the WR treatment (*W* = 45.0, *p* < 0.001) (Figure 1). Under LC_25_ indoxacarb exposure, the difference in developmental duration between RR and WW was no longer significant, whereas larvae in the RR treatment showed significantly longer developmental duration than those in the RW treatment (*W* = 451.0, *p* < 0.001) (Figure 1). LC_25_ indoxacarb significantly prolonged developmental duration in RR (*W* = 96.5, *p* < 0.001) and WW larvae (*W* = 148.5, *p* < 0.001) but shortened it in the WR treatment (*W* = 410.5, *p* = 0.046) (Figure 1).

For the 4th instar, LC_25_ indoxacarb treatment and its interaction with population origin significantly influenced developmental duration, while population origin, host switching treatment, and the other interactions were not significant (Table 3). LC_25_ indoxacarb significantly prolonged developmental duration in all four host switching treatments (RR: *W* = 130.5, *p* = 0.008; RW: *W* = 434.5, *p* = 0.002; WW: *W* = 28.0, *p* < 0.001; WR: *W* = 78.0, *p* < 0.001) (Figure 1). In the control groups, larvae in the RR treatment exhibited significantly longer developmental duration than those in the WW treatment (*W* = 450.0, *p* = 0.025). However, after LC_25_ indoxacarb exposure, larvae in the RR treatment showed significantly shorter developmental duration than those in the WW treatment (*W* = 138.0, *p* = 0.003), and larvae in the RW treatment also showed significantly shorter developmental duration than those in the WR treatment (*W* = 124.0, *p* < 0.001) (Figure 1). These treatment-dependent patterns indicate that the effect of host switching on 4th-instar developmental duration depends on insecticide exposure, which is consistent with the significant interaction between insecticide treatment and population origin.

For the 5th instar, LC_25_ indoxacarb treatment and population origin showed significant effects, while host switching treatment did not. The interactions between insecticide treatment and population origin, between population origin and host switching treatment, and among insecticide treatment, population origin, and host switching treatment were all significant (Table 3). In the control groups, larvae in the RR treatment exhibited significantly longer developmental duration than those in the WW treatment (*W* = 485.5, *p* = 0.003), and larvae in the RW treatment exhibited significantly shorter developmental duration than those in the WR treatment (*W* = 25.0, *p* < 0.001) (Figure 1). RW and WW treatments exhibited significantly shorter developmental duration than the RR (RW-RR: *W* = 736.0, *p* < 0.001) and WR (WR-WW: *W* = 762.5, *p* < 0.001) treatments, respectively. Under LC_25_ indoxacarb exposure, larvae in the RR treatment showed significantly longer duration than those in the RW treatment (*W* = 566.5, *p* = 0.017), whereas the difference in developmental duration between RW and WR was no longer significant (Figure 1). LC_25_ indoxacarb significantly prolonged developmental duration in RR, WW, and RW treatments (RR: *W* = 36.0, *p* < 0.001; WW: *W* = 173.0, *p* < 0.001; RW: *W* = 25.2, *p* < 0.001) (Figure 1).

These results suggest that the effects of population origin, host switching, and sublethal indoxacarb exposure on larval developmental duration are instar-specific. LC_25_ indoxacarb eliminated the RR-WW difference in the 3rd instar and the RW-WR difference in the 5th instar, whereas in the 4th instar its effect depended on population origin. Overall, host background modulated the sublethal effects of indoxacarb in an instar-specific manner.

### 3.3. Sublethal Effects of Indoxacarb on Pupation, and Fecundity of C. medinalis Under Different Host Switching Treatments

**Figure 2:**
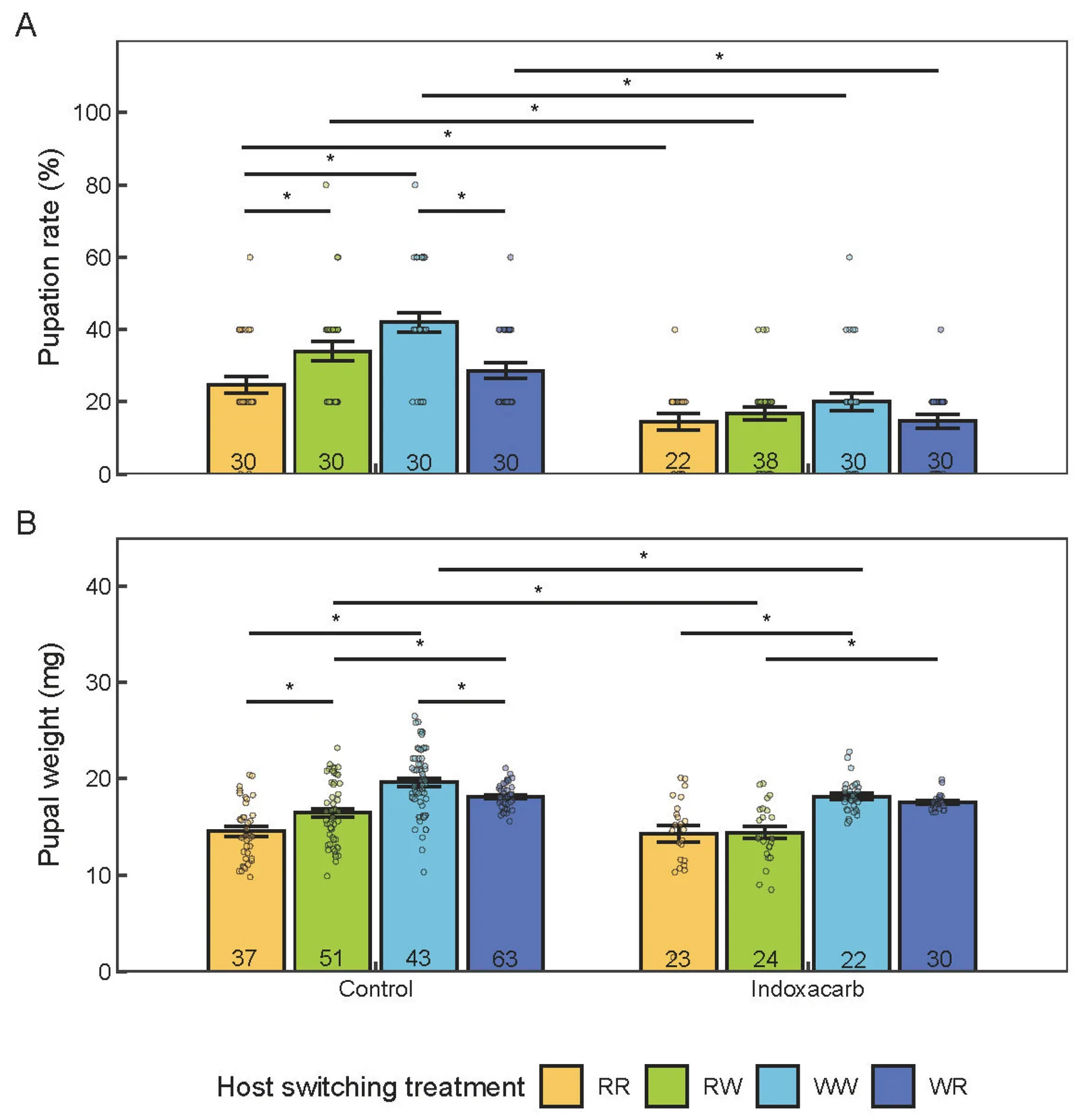
Pupation rate (**A**) and pupal weight (**B**) of *C. medinalis* under LC_25_ indoxacarb exposure and different host switching backgrounds. Dots represent individual sample values. Bars represent means, and error bars represent standard errors (mean ± SE). The numbers on the bars indicate sample sizes. Asterisks indicate significant differences among different treatment groups (Mann–Whitney U test-*p* < 0.05). Host switching abbreviations: RR, rice-population reared on rice; RW, rice-population reared on wheat; WR, wheat-population reared on rice; WW, wheat-population reared on wheat.

Scheirer–Ray–Hare test showed that pupation rate was significantly affected by insecticide treatment, but not by population origin or host switching treatment; the interaction between population origin and host switching treatment was significant, whereas the remaining interactions were not (Table 4). Pupal weight was significantly affected by insecticide treatment and population origin, but not by host switching treatment; the interaction between population origin and host switching treatment was significant, whereas the remaining interactions were not (Table 4). In the control groups, larvae in the RR treatment exhibited significantly lower pupation rate than those in the WW treatment (*W* = 183.5, *p* < 0.001) (Figure 2). Similarly, pupal weight in the RR treatment was significantly lower than that in the WW treatment (*W* = 316.0, *p* < 0.001), and pupal weight in the RW treatment was significantly lower than that in the WR treatment (*W* = 743.5, *p* < 0.001) (Figure 2). In addition, larvae in the RR treatment exhibited significantly lower pupation rate (*W* = 298.0, *p* = 0.012), and pupal weight (*W* = 640.0, *p* = 0.010) than those in the RW treatment, while larvae in the WW treatment showed significantly higher pupation rate (*W* = 233.5, *p* < 0.001), and pupal weight (*W* = 917.0, *p* = 0.005) than the WR treatment (Figure 2). Under LC_25_ indoxacarb exposure, the difference in pupation rate between RR and WW was no longer significant, whereas the differences in pupal weight between RR and WW (*W* = 128.5, *p* < 0.001) and between RW and WR remained (*W* = 89.5, *p* < 0.001) significant (Figure 2). When comparing insecticide effects within the same host switching background, LC_25_ indoxacarb significantly reduced pupation rate (RW: *W* = 911.0, *p* < 0.001; WW: *W* = 761.5, *p* < 0.001), and pupal weight (RW: *W* = 804.5, *p* = 0.029; WW: *W* = 1230.5, *p* = 0.019) in RW and WW treatments (Figure 2). In RR and WR treatments, LC_25_ indoxacarb significantly decreased pupation rates (RR: *W* = 459.5, *p* = 0.005; WR: *W* = 6965, *p* < 0.001), but did not significantly affect pupal weight (Figure 2).

**Figure 3:**
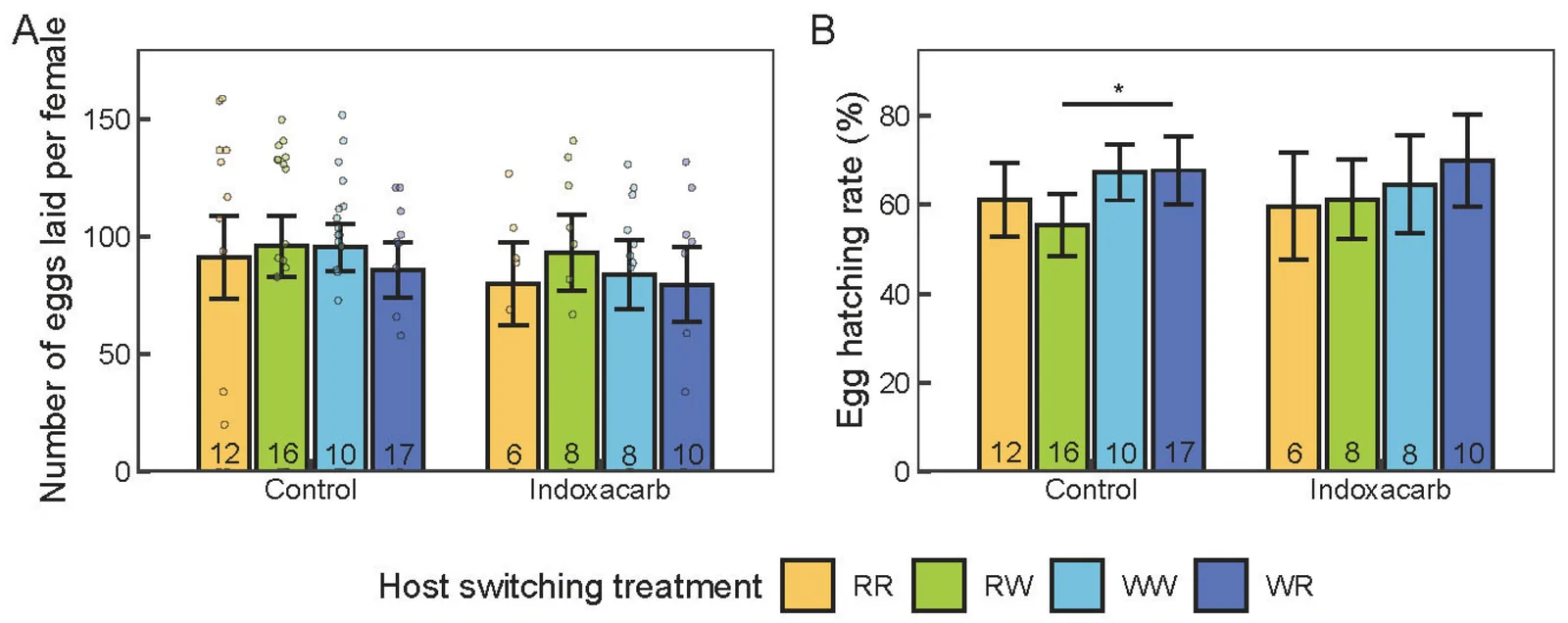
Number of eggs laid per female and egg hatching rate of *C. medinalis* under LC_25_ indoxacarb exposure and different host switching backgrounds. Dots represent individual sample values. Bars represent means, and error bars represent standard errors (mean ± SE). The numbers on the bars indicate sample sizes. Asterisks indicate significant differences among different treatment groups (Mann–Whitney U test-*p* < 0.05). Host switching abbreviations: RR, rice-population reared on rice; RW, rice-population reared on wheat; WR, wheat-population reared on rice; WW, wheat-population reared on wheat.

Number of eggs laid per female was not significantly affected by insecticide treatment, population origin, host switching treatment, or their interactions (Table 4). Egg hatching rate was significantly affected by population origin, but not by insecticide treatment, host switching treatment, or their interactions (Table 4). In the control groups, egg hatching rate in the RW treatment was significantly lower than that in the WR treatment (*W* = 459.5, *p* = 0.004) (Figure 3). However, after LC_25_ indoxacarb exposure, the difference in egg hatching rate between RW and WR was no longer significant (Figure 3). Within the same population origin, neither host switching treatment nor LC_25_ indoxacarb exposure significantly affected number of eggs laid per female or egg hatching rate (Figure 3). These results indicate that sublethal indoxacarb mainly affected pupal traits, whereas reproductive traits were less responsive to insecticide exposure and were more influenced by population origin.

### 3.4. Sublethal Effects of Indoxacarb on Expression of P450-Related Genes in C. medinalis Under Different Host Switching Treatments

Three-way ANOVA showed that the expression of P450-related genes was differentially affected by insecticide treatment, population origin, host switching treatment, and their interactions (Table 5). Insecticide treatment significantly affected *CmedCYP49A1*, *CmedCYP12A2*, *CmedCYP301A1*, *CmedCYP4AE76*, and *CmedCYP4V2*, but not *CmedCYP6AE30* (Table 5). Population origin significantly affected *CmedCYP12A2*, but not the other five P450 genes (Table 5). Host switching treatment significantly affected *CmedCYP49A1* and *CmedCYP4AE76*, but not *CmedCYP6AE30*, *CmedCYP12A2*, *CmedCYP301A1*, or *CmedCYP4V2* (Table 5). The interaction between insecticide treatment and population origin was significant for *CmedCYP6AE30*, *CmedCYP301A1*, and *CmedCYP4AE76* (Table 5). The interaction between insecticide treatment and host switching treatment was significant for *CmedCYP301A1* (Table 5). The interaction between population origin and host switching treatment was not significant for any P450 gene (Table 5). The three-way interaction among insecticide treatment, population origin, and host switching treatment was significant for *CmedCYP6AE30* (Table 5).

**Figure 4:**
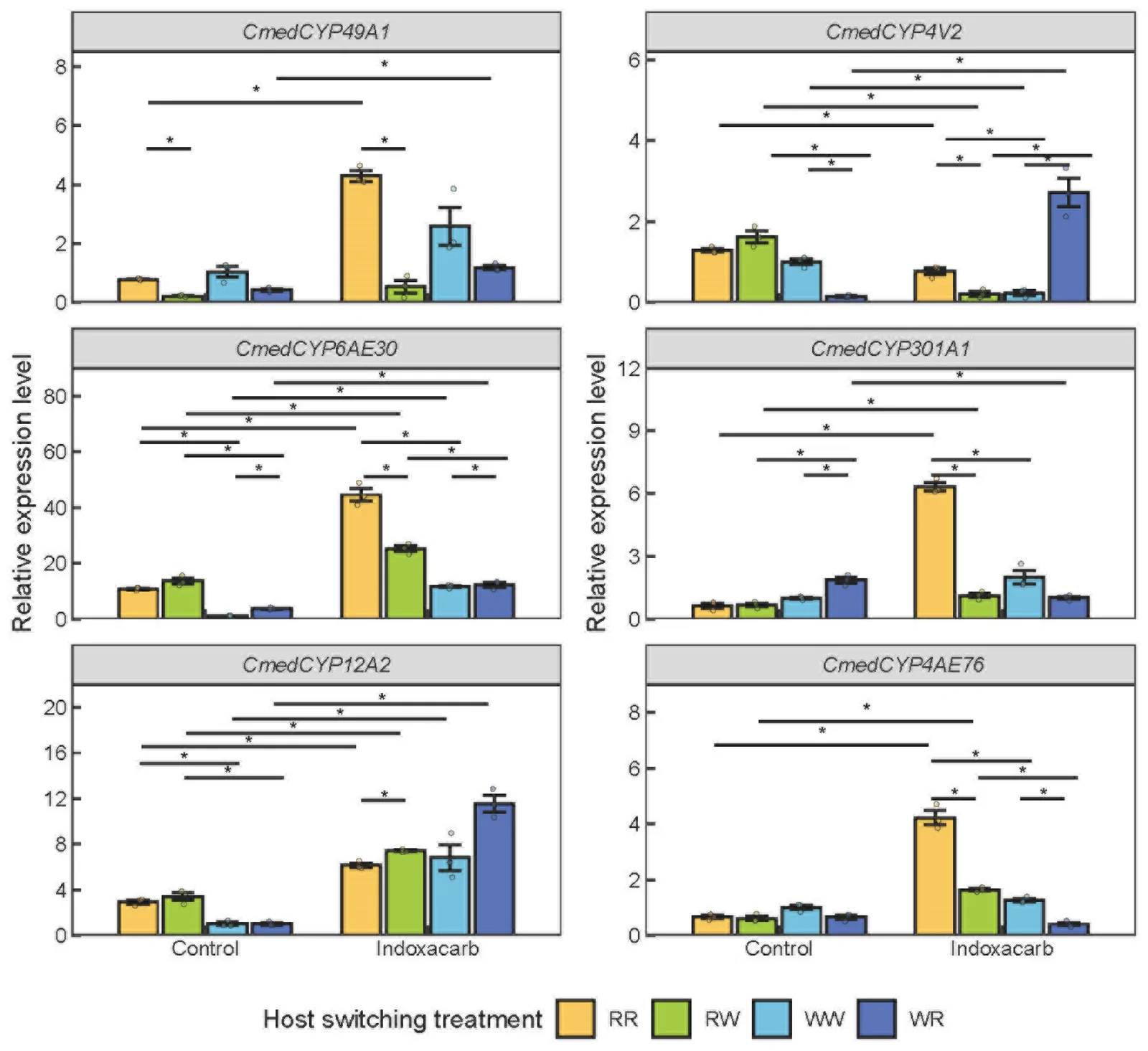
Expression profiles of P450-related genes in *C. medinalis* under LC_25_ indoxacarb exposure and different host switching backgrounds. Dots represent individual sample values. Bars represent means, and error bars represent standard errors (mean ± SE). Asterisks indicate significant differences among different treatment groups (*t* test-*p* < 0.05). Host switching abbreviations: RR, rice-population reared on rice; RW, rice-population reared on wheat; WR, wheat-population reared on rice; WW, wheat-population reared on wheat.

When comparing insecticide effects within the same host switching background, LC_25_ indoxacarb exposure significantly upregulated *CmedCYP49A1* (*t* = 19.8423, df = 2.049, *p* = 0.002), *CmedCYP6AE30* (*t* = 5.412, df = 3.242, *p* = 0.010), *CmedCYP12A2* (*t* = 14.300, df = 2.082, *p* = 0.004), *CmedCYP301A1* (*t* = 24.722, df = 3.281, *p* < 0.001), and *CmedCYP4AE76* (*t* = 13.721, df = 2.218, *p* = 0.003) in the RR treatment, while *CmedCYP4V2* was significantly downregulated (*t* = 12.388, df = 3.986, *p* < 0.001) (Figure 4). In the RW treatment, LC_25_ indoxacarb significantly upregulated *CmedCYP6AE30* (*t* = 8.983, df = 2.600, *p* = 0.001), *CmedCYP12A2* (*t* = 7.880, df = 3.997, *p* = 0.001), *CmedCYP301A1* (*t* = 3.116, df = 3.822, *p* = 0.038), and *CmedCYP4AE76* (*t* = 10.760, df = 3.130, *p* = 0.001), but downregulated *CmedCYP4V2* (*t* = 11.445, df = 2.198, *p* = 0.005) (Figure 4). In the WW treatment, LC_25_ indoxacarb significantly upregulated *CmedCYP6AE30* (*t* = 8.084, df = 3.734, *p* = 0.001) and *CmedCYP12A2* (*t* = 26.715, df = 2.552, *p* < 0.001), while *CmedCYP4V2* was significantly downregulated (*t* = 5.050, df = 2.060, *p* = 0.035) (Figure 4). In the WR treatment, LC_25_ indoxacarb significantly upregulated *CmedCYP49A1* (*t* = 8.806, df = 3.240, *p* = 0.002), *CmedCYP6AE30* (*t* = 7.326, df = 2.008, *p* = 0.018), *CmedCYP12A2* (*t* = 5.525, df = 3.098, *p* = 0.012), and *CmedCYP4V2* (*t* = 14.456, df = 2.073, *p* = 0.004), but significantly downregulated *CmedCYP301A1* (*t* = 5.325, df = 3.098, *p* = 0.012) (Figure 4).

Beyond the effects of LC_25_ indoxacarb within the same host background, host background also led to significant differences in the expression of the six P450 genes. In the control groups, the RR treatment exhibited significantly lower expression of *CmedCYP49A1* than the RW treatment (*t* = 20.591, df = 4.000, *p* < 0.001) (Figure 4). For *CmedCYP6AE30*, expression in the control groups was significantly higher in RR than in WW (*t* = 27.109, df = 2.677, *p* < 0.001) and significantly lower in RW than in WR (*t* = 9.464, df = 2.162, *p* = 0.008), and this pattern persisted after LC_25_ indoxacarb exposure (RR-WW: *t* = 13.928, df = 2.101, *p* = 0.004; WR-WR: *t* = 9.583, df = 3.818, *p* < 0.001) (Figure 4). For *CmedCYP12A2*, expression in the control groups was significantly higher in RR than in WW (*t* = 8.475, df = 3.790, *p* = 0.001) and in RW than in WR (*t* = 6.807, df = 2.327, *p* = 0.013), but these differences disappeared after LC_25_ indoxacarb exposure (Figure 4). Meanwhile, compared with the WW treatment, the WR treatment showed significantly higher expression of *CmedCYP6AE30* (*t* = 10.984, df = 2.180, *p* = 0.006) and *CmedCYP301A1* (*t* = 5.810, df = 2.485, *p* = 0.017), but significantly lower expression of *CmedCYP4V2* (*t* = 10.766, df = 3.509, *p* = 0.005) (Figure 4). For *CmedCYP301A1*, expression in the control groups was significantly lower in RW than in WR (*t* = 7.111, df = 3.462, *p* = 0.003); this difference disappeared after LC_25_ indoxacarb exposure, whereas RR showed significantly higher expression than WW under LC_25_ indoxacarb exposure (*t* = 11.580, df = 3.344, *p* < 0.001) (Figure 4). For *CmedCYP4V2*, expression in the control groups was significantly higher in RW than in WR (*t* = 10.003, df = 2.048, *p* = 0.009), with no significant difference between RR and WW; after LC_25_ indoxacarb exposure, RR showed significantly higher expression than WW (*t* = 5.282, df = 3.566, *p* = 0.008) (Figure 4). Under LC_25_ indoxacarb exposure, the RR treatment showed significantly higher expression of *CmedCYP49A1* (*t* = 13.566, df = 3.856, *p* < 0.001), *CmedCYP6AE30* (*t* = 5.492, df = 3.558, *p* = 0.007), *CmedCYP4V2* (*t* = 6.184, df = 2.630, *p* = 0.012), *CmedCYP301A1* (*t* = 22.859, df = 3.200, *p* < 0.001), and *CmedCYP4AE76* (*t* = 10.045, df = 2.133, *p* = 0.008) than the RW treatment, while *CmedCYP12A2* showed the opposite pattern (Figure 4). After LC_25_ indoxacarb exposure, *CmedCYP4AE76* expression was significantly higher in RR than in WW (*t* = 11.367, df = 2.204, *p* = 0.005) and in RW than in WR (*t* = 16.366, df = 3.774, *p* < 0.001) (Figure 4). The WR treatment showed significantly higher expression of *CmedCYP6AE30* (*t* = 7.007, df = 2.109, *p* = 0.017) and *CmedCYP4V2* (*t* = 3.499, df = 3.382, *p* = 0.033) than the RW treatment, but significantly lower expression of *CmedCYP4AE76* (*t* = 10.484, df = 43.995, *p* = 0.004) (Figure 4). These results suggest that population origin and host switching differentially affected P450 expression in a gene-specific manner, and these effects were modulated by LC_25_ indoxacarb exposure, with some host-associated differences persisting after treatment and others disappearing or emerging.

### 3.5. Sublethal Effects of Indoxacarb on Expression of Juvenile Hormone-Related Genes in C. medinalis Under Different Host Switching Treatments

Three-way ANOVA revealed that the four JH-related genes were differentially affected by insecticide treatment, population origin, host switching treatment, and their interaction on all four JH-related genes (Table 5). Insecticide treatment significantly affected *CmedJHAMT*, *CmedJHE*, and *CmedJHBP1*, but not *CmedAT*. Population origin significantly affected all four JH-related genes (*CmedAT*, *CmedJHAMT*, *CmedJHE*, and *CmedJHBP1*). Host switching treatment significantly affected *CmedAT* and *CmedJHAMT*, but not *CmedJHE* or *CmedJHBP1*. None of the two-way or three-way interactions were significant for any of the four JH-related genes (Table 5).

**Figure 5:**
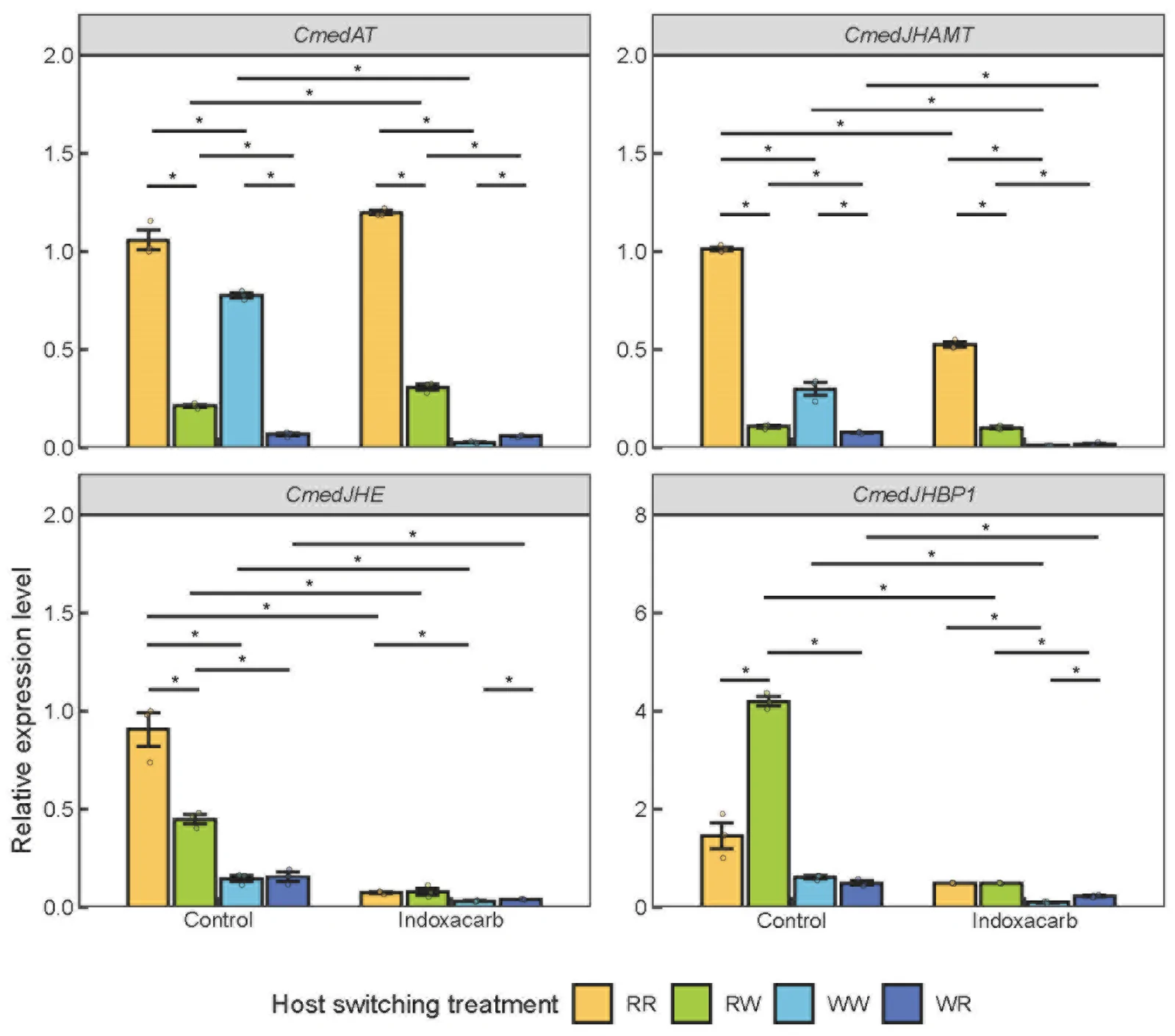
Expression profiles of JH-related genes in *C. medinalis* under LC_25_ indoxacarb exposure and different host switching backgrounds. Dots represent individual sample values. Bars represent means, and error bars represent standard errors (mean ± SE). Asterisks indicate significant differences among different treatment groups (*t* test-*p* < 0.05). Host switching abbreviations: RR, rice-population reared on rice; RW, rice-population reared on wheat; WR, wheat-population reared on rice; WW, wheat-population reared on wheat.

LC_25_ indoxacarb exposure significantly upregulated *CmedAT* in the RW treatments (*t* = 5.791, df = 2.977, *p* = 0.010), but downregulated it in the WW treatments (*t* = 56.362, df = 2.176, *p* < 0.001) (Figure 5). *CmedJHAMT* was significantly downregulated by LC_25_ indoxacarb in the RR (*t* = 30.962, df = 3.700, *p* < 0.001), WW (*t* = 9.057, df = 2.593, *p* = 0.005), and WR (*t* = 8.849, df = 2.001, *p* = 0.049) treatments (Figure 5). CmedJHBP1 expression was significantly downregulated by LC_25_ indoxacarb in the RW (*t* = 38.762, df = 2.018, *p* < 0.001), WW (*t* = 6.026, df = 2.729, *p* < 0.001), and WR (*t* = 6.026, df = 2.542, *p* = 0.014) treatments, but not in the RR treatment (Figure 5). In all four host switching treatments, LC_25_ indoxacarb significantly reduced the expression of *CmedJHE* (RR: *t* = 9.816, df = 2.011, *p* = 0.010; RW: *t* = 12.571, df = 3.642, *p* < 0.001; WW: *t* = 7.098, df = 2.049, *p* = 0.018; WR: *t* = 5.162, df = 2.006, *p* = 0.035) compared with their respective controls (Figure 5).

In the control groups, the RR treatment showed significantly higher expression of *CmedAT* (*t* = 16.855, df = 2.092, *p* < 0.001), *CmedJHAMT* (*t* = 80.313, df = 3.446, *p* < 0.001), and *CmedJHE* (*t* = 5.203, df = 2.314, *p* = 0.035) than the RW treatment, while *CmedJHBP1* (*t* = 9.925, df = 2.533, *p* = 0.001) showed the opposite trend (Figure 5). Compared with WW, RR showed significantly higher expression of *CmedAT* (*t* = 5.506, df = 2.273, *p* = 0.034), *CmedJHAMT* (*t* = 21.003, df = 2.333, *p* = 0.001), and *CmedJHE* (*t* = 8.8301, df = 2.143, *p* = 0.016) (Figure 5). In addition, RW showed significantly higher expression of all four JH-related genes than WR (*CmedAT*: *t* = 13.503, df = 4.000, *p* < 0.001; *CmedJHAMT*: *t* = 4.629, df = 2.593, *p* = 0.035; *CmedJHE*: *t* = 8.831, df = 2.143, *p* = 0.010; *CmedJHBP1*: *t* = 35.618, df = 2.727, *p* < 0.001) (Figure 5). The WW treatment exhibited significantly higher expression of *CmedAT* (*t* = 46.957, df = 3.234, *p* < 0.001), *CmedJHAMT* (*t* = 6.821, df = 2.022, *p* = 0.031) than the WR treatment, with no significant differences in *CmedJHE* or *CmedJHBP1* (Figure 5). Under indoxacarb exposure, the RR treatment showed significantly higher expression of *CmedAT* (*t* = 48.995, df = 3.647, *p* < 0.001) and *CmedJHAMT* (*t* = 30.621, df = 2.718, *p* < 0.001) than the RW treatment (Figure 5). In addition, *CmedJHBP1* expression in RR was significantly higher than that in WW (Figure 5). The WR treatment exhibited significantly higher expression of *CmedAT* (*t* = 8.332, df = 3.996, *p* = 0.001), *CmedJHBP1* (*t* = 6.418, df = 3.922, *p* = 0.003) and *CmedJHE* (*t* = 4.564, df = 2.941, *p* = 0.031) than the WW treatment (Figure 5). However, the significant difference in *CmedJHE* expression between RW and WR observed in the control groups (*t* = 9.010, df = 3.982, *p* < 0.001) disappeared after LC_25_ indoxacarb exposure (Figure 5). These results suggest that the four JH-related genes differed in their responses to population origin and host switching, and these host-associated expression patterns were further altered by LC_25_ indoxacarb exposure in a gene-dependent manner.

### 3.6. Correlations Among P450- and JH-Related Gene Expression Levels and Biological Parameters of C. medinalis Under Sublethal Indoxacarb Exposure and Different Host Switching Backgrounds

**Figure 6:**
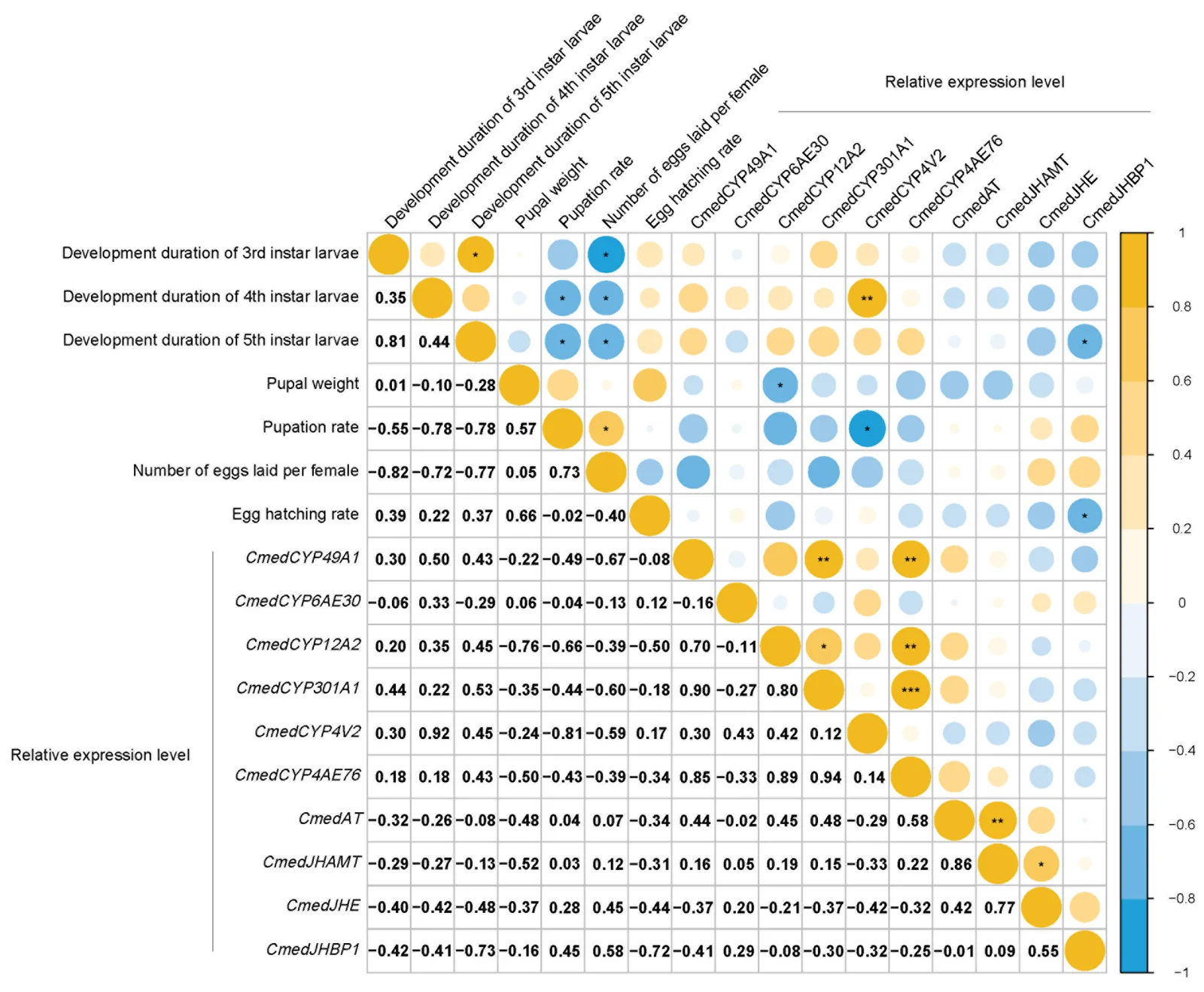
Spearman correlation analysis of the expression levels of P450- and JH-related genes and key biological parameters of *C. medinalis* under sublethal indoxacarb exposure and different host switching backgrounds. The numbers in the bottom left corner represent the correlation coefficient (*r*). *, **, and *** indicate significant correlations at *p* < 0.05, *p* < 0.01, and *p* < 0.001, respectively. The darker the yellow color, the stronger the positive correlation, while the darker the blue color, the stronger the negative correlation. A larger circle indicates a smaller *p*-value.

Spearman correlation analysis revealed that gene expression levels and biological parameters were associated (Figure 6). Among biological parameters, the developmental duration of 3rd-instar larvae showed a significant positive correlation with that of 5th-instar larvae, but a significant negative correlation with number of eggs laid per female (Figure 6). The developmental durations of both 4th- and 5th-instar larvae were significantly negatively correlated with pupation rate and fecundity (Figure 6).

Regarding the correlations between gene expression and biological parameters, the developmental duration of fourth-instar larvae was significantly positively correlated with the expression of *CmedCYP4V2* (Figure 6). The developmental duration of 5th-instar larvae was significantly negatively correlated with the expression of *CmedJHBP1* (Figure 6). Pupal weight was significantly negatively correlated with the expression of *CmedCYP12A2* (Figure 6). Pupation rate was significantly negatively correlated with the expression of *CmedCYP4V2* (Figure 6). Egg hatchability was significantly negatively correlated with the expression of *CmedJHBP1* (Figure 6).

In terms of gene-gene correlations, the expression of *CmedCYP49A1* and *CmedCYP12A2* was significantly positively correlated with the expression of *CmedCYP301A1* and *CmedCYP4AE76* (Figure 6). The expression of *CmedCYP301A1* was significantly positively correlated with that of *CmedCYP4AE76* (Figure 6). Additionally, *CmedAT* and *CmedJHAMT* showed a significant positive correlation, as did *CmedJHAMT* and *CmedJHE* (Figure 6).

## 4. Discussion

Host plant switching can induce physiological plasticity in polyphagous insects and may alter their susceptibility to insecticides through the induction or suppression of detoxification enzyme systems [1,34]. Previous studies in *C. medinalis* have shown that host switching can modify its susceptibility to abamectin and chlorpyrifos, as well as the sublethal effects of these insecticides [30,35]. In this study, we examined the effects of insecticide treatment, population origin, and host switching on life-history traits and gene expression. The results indicate that these factors interact in a trait- and instar-specific manner. The expression patterns of P450 and JH-related genes varied across host backgrounds and were correlated with certain phenotypic changes.

Host switching significantly altered the susceptibility of *C. medinalis* larvae to indoxacarb. The RR treatment showed the highest susceptibility, whereas the WW treatment exhibited the lowest. The RW and WR treatments showed intermediate levels, with WR partially restoring susceptibility but not to the RR level, suggesting that host switching can partly reverse resistance acquired through long-term adaptation. This pattern suggests that host switching can shift the expressed susceptibility phenotype, but it does not necessarily indicate reversal of resistance mechanisms acquired through long-term adaptation. This pattern is similar to that of chlorpyrifos but differs from abamectin. For chlorpyrifos, the WR treatment also resulted in higher susceptibility than the WW treatment, whereas host switching decreased susceptibility to abamectin in both populations [30]. These differences may be attributed to their distinct modes of action and metabolic pathways. Indoxacarb acts on sodium channels [29], abamectin targets chloride channels [36], and chlorpyrifos inhibits acetylcholinesterase [37]. In addition, host plants can induce different detoxification enzymes, including P450s, which may vary in their efficiency in metabolizing the three insecticides [9]. Host plants may regulate P450 and other detoxification genes, thereby influencing the insect’s capacity to metabolize different insecticides [38]. The effect of host switching on susceptibility can change with host background, and its direction and magnitude depend on the host plant-insecticide combination [30]. Previous studies have shown that the rate of resistance evolution is closely related to host range and the chemical similarity between insecticides and plant defensive compounds, and that polyphagous insects may have greater potential for resistance evolution due to their evolutionary history of coping with diverse plant allelochemicals [1,2]. However, the two populations differ in collection location and rearing history, so population origin is partially confounded with long-term host experience. These differences in indoxacarb susceptibility are therefore more likely to reflect host-associated shifts in susceptibility phenotype than a reversal of resistance mechanisms. In the WR treatment, switching from wheat to rice partially restored susceptibility, demonstrating that the host-insecticide adaptation relationship can change when the host background is altered. This suggests that the link between host plants and resistance is not fixed but can shift in response to host switching. This means that the host plant history of pest populations should be considered when selecting and applying different types of insecticides.

Both host plants and sublethal insecticides can influence insect growth, development, and reproduction [7,39]. In our study, larval developmental duration was affected by insecticide treatment, population origin, host switching, and their interactions in an instar-specific manner. In the 3rd instar, LC_25_ indoxacarb eliminated the difference between RR and WW; in the 4th instar, insecticide treatment prolonged development in all treatments, and its effect depended on population origin; in the 5th instar, LC_25_ indoxacarb eliminated the difference between RW and WR but not between RR and RW. Overall, host background modulated the sublethal effects of indoxacarb in an instar-specific manner. In *C. medinalis*, the 3rd instar is characterized by intensive feeding and high metabolic activity, whereas the 5th instar represents a critical window for metamorphosis initiation [40]. This difference may be related to stage-specific JH activity [17]. Our previous work with abamectin and chlorpyrifos similarly showed that LC_25_ exposure significantly prolonged the total developmental duration from the 3rd to 5th instar, but the extent of prolongation depended on host switching history [35]. In most insects, sublethal insecticide exposure tends to prolong larval developmental duration, but the effects are not consistent across different species. For example, broflanilide at LC_25_ significantly prolonged the larval developmental duration of *Spodoptera litura* [41]; sublethal indoxacarb exposure shortened the larval developmental time in the parental generation of *Spodoptera frugiperda* but prolonged it in the F_1_ generation [42]. In our study, LC_25_ indoxacarb significantly shortened the developmental duration of 3rd instar larvae in the WR treatment, which may reflect a stress-induced escape response, in which larvae accelerate development under moderate stress to reduce exposure time [8]. For pupal traits, pupation rate was significantly affected by insecticide treatment and by the interaction between population origin and host switching, whereas pupal weight was significantly affected by insecticide treatment and population origin. In the control groups, RR exhibited lower pupation rate and pupal weight than WW, and RW exhibited lower pupal weight than WR. After LC_25_ indoxacarb exposure, the difference in pupation rate between RR and WW disappeared, whereas the differences in pupal weight between RR and WW and between RW and WR remained significant. These results differs from the effects of abamectin and chlorpyrifos [35], suggesting that the impact of sublethal insecticides on pupal traits depends on the insecticide type and host background. Notably, neither insecticide treatment, population origin, host switching, nor their interactions significantly affected the number of eggs laid per female. Egg hatching rate was significantly affected by population origin, but not by insecticide treatment, host switching, or their interactions. In the control groups, egg hatching rate in RW was significantly lower than that in WR, but this difference disappeared after LC_25_ indoxacarb exposure. These results indicate that sublethal indoxacarb mainly affected pupal traits, whereas reproductive traits were less responsive to insecticide exposure and were more influenced by population origin. Insects may prioritize reproductive output under sublethal stress or host changes to compensate for reductions in individual fitness. The relative stability of reproductive traits may represent an important strategy for many insects to sustain population continuation under unfavorable conditions.

The cytochrome P450 monooxygenase system is a core detoxification system for insects responding to plant defenses and insecticide stress [9]. In this study, P450 genes expression was differentially affected by insecticide treatment, population origin, host switching, and their interactions, and the effects were gene-specific. Among the six P450 examined, *CmedCYP6AE30* and *CmedCYP301A1* showed particularly strong interactive effects, whereas *CmedCYP12A2* was mainly affected by population origin and *CmedCYP49A1* and *CmedCYP4AE76* by host switching. In other insects, members of the CYP6AE subfamily are widely involved in xenobiotic metabolism and insecticide detoxification; for example, *CYP6AE43* in *Spodoptera frugiperda* participates in chlorantraniliprole metabolism [11]. *CYP301A1* is a highly conserved P450 gene in insects, involved in ecdysone regulation and cuticle formation in *Drosophila* [43]. These findings suggest that *CmedCYP6AE30* and *CmedCYP301A1* may be candidate genes linking host adaptation and insecticide response in *C. medinalis*, but functional validation is required. In addition, *CYP49A1* is highly correlated with chlorantraniliprole resistance in *S. frugiperda* [44]; overexpression of *CYP12A2* in *Plutella xylostella* mediates metabolic resistance to pyridalyl, and RNAi knockdown of *CYP12A2* significantly increases larval susceptibility to pyridalyl [45]. Correlation analysis further revealed that *CmedCYP12A2* expression was significantly negatively correlated with pupal weight, while *CmedCYP4V2* expression was positively correlated with 4th instar duration and negatively correlated with pupation rates. Moreover, *CmedCYP49A1* and *CmedCYP12A2* showed significant positive correlations with *CmedCYP301A1* and *CmedCYP4AE76* expression. These associations suggest that P450 genes may be involved in developmental regulation and insecticide response. In addition to their detoxification function, P450 enzymes also participate in the synthesis and metabolism of endogenous hormones, suggesting a possible functional link between the P450 system and the JH pathway. In *Tribolium castaneum*, the CncC signaling pathway has been found to simultaneously regulate P450 detoxification genes and JH degradation enzymes, mediating the trade-off between detoxification and development [20]. Sublethal insecticides similarly affected JH pathway gene expression and altered JH titers, as reported in *Chilo suppressalis* and *Nilaparvata lugens* [15,16]. In this study, JH-related gene expression was also differentially affected by insecticide treatment, population origin, and host switching, but the interactions among these factors were not significant, indicating that insecticide and host factors largely acted independently on JH-related gene expression. LC_25_ indoxacarb exposure significantly upregulated *CmedAT* in the RW treatment but downregulated in the WW treatment, suggesting that the effect of indoxacarb on JH synthesis depends on host background. *CmedJHAMT* was significantly downregulated by LC_25_ indoxacarb in the RR, WW, and WR treatments. *CmedJHE* was coordinately downregulated across all four treatments, suggesting that indoxacarb may suppress JH catabolism, which could explain the prolonged developmental duration observed in most treated groups. *CmedJHBP1* was significantly downregulated by LC_25_ indoxacarb in the RW, WW, and WR treatments, but not in the RR treatment, indicating that JH signal transduction may be suppressed. However, correlation analysis revealed no significant correlations between P450 and JH-related gene expression. Several explanations for this result are possible. First, coordinated responses between the P450 and JH pathways may be time-dependent [46,47], and our measurements at a single time point (48 h) may not have captured transcriptional co-regulation that occurs at earlier or later time windows. Second, regulation between the P450 system and the JH pathway may occur primarily at the post-translational level or at the level of enzyme activity rather than through direct transcriptional co-regulation [48,49]. Third, the Spearman correlation analysis was exploratory, and the treatment combinations may influence the observed correlations. These possibilities warrant further investigation through multi-time-point monitoring and functional validation experiments.

## 5. Conclusions

In conclusion, this study indicates that host switching significantly alters larval susceptibility to indoxacarb, and that host pre-adaptation and sublethal indoxacarb exposure interactively affect the growth and development of the migratory pest *C. medinalis*. Furthermore, the expression of P450 and juvenile hormone-related genes is differentially regulated by host background and insecticide exposure, and these expression patterns are correlated with certain phenotypic changes. However, these results only demonstrate an association between gene expression changes and the observed effects; they do not establish the underlying mechanisms. Because gene expression was measured at a single time point and no functional validation was performed, the causal roles of these genes in mediating the observed phenotypic effects remain unproven. Based on these associations, we propose the hypothesis that P450 detoxification genes and juvenile hormone-related genes may participate in the metabolic and hormonal remodeling associated with host plant adaptation and insecticide stress responses in this migratory pest. Understanding the interplay between host plant history, sublethal insecticide stress, and the expression of key P450 and juvenile hormone genes may provide a theoretical basis for optimizing indoxacarb use and managing resistance risk in the field. Nevertheless, further validation of causal relationships and elucidation of molecular mechanisms are required through experiments such as RNA interference and functional gene analysis before such knowledge can be translated into practical applications.

## Figures and Tables

**Table 1:** Primer information.

Primer Name	Gene ID	Sequence of Primers (5′-3′)	Primer Use
*CmedCYP49A1*	Cmed124040	F: CATCATGCCCAGTTCACGGAC R: GCACCTCCTCCCAAGACCTCA	RT-qPCR
*CmedCYP6AE30*	Cmed007000	F: TGCCTGTAACTGACGACT R: TCATATGTTAGCTGGTTTCCG	RT-qPCR
*CmedCYP* *12A2*	Cmed105690	F: AAACAAGACAAACTAAGGGAG R: GGTATCTTTAGGTATAGCGTA	RT-qPCR
*CmedCYP301A* *1*	Cmed012840	F: CGTTGAACTCTGCTCGCGCTA R: ACAGACCGCCATTGAAATCGT	RT-qPCR
*CmedCYP* *4V2*	Cmed046830	F: TTCAACCCAGAGAACACGAAGC R: TGGATGACACCTCGAACTCCC	RT-qPCR
*CmedCYP4AE76*	Cmed006990	F: TGCCGTTCGTCAAGCCTA R: CATAGAAAGCGCCCACGTA	RT-qPCR
*CmedAT*	Cmed126120	F: TGTTTTGTCTCCAGCATCCCC R: CAACAAACTTGCGGACGACA	RT-qPCR
*CmedJHAMT*	Cmed134230	F: AACGCTGAACTCTACCAAGGAA R: TTCTCAAAGTTATCGGGCAGGT	RT-qPCR
*CmedJHE*	Cmed124590	F: CGACGACATCACAGTGGTTA R: CGTTGACTGGATTCGCTTTG	RT-qPCR
*CmedJHBP1*	Cmed070120	F: TGGGAACTGCAAAGTACAACT R: GCCCAAAGCCTCACAAGTAA	RT-qPCR
*Cmedβ* *-* *actin*	JN029806.1	F: CACACAGTGCCCATCTACGA R: GCGGTGGTGGTGAATGAGTA	Reference gene

**Table 2:** Toxicity of indoxacarb to *Cnaphalocrocis medinalis* larvae of different host switching treatments.

Treatment	LC_25_ (95% Confidence Intervals) (mg/L)	LC_50_ (95% Confidence Intervals) (mg/L)	Toxicity Regression Equation	Relative Toxicity
Rice populations-rice (RR)	0.001 (0.000~0.002)	0.016 (0.006~0.033)	y = 1.319 + 0.734x	1.00
Rice populations-wheat (RW)	0.012 (0.005~0.021)	0.115 (0.066~0.306)	y = 1.057 + 1.127x	2.37
Wheat populations-wheat (WW)	0.064 (0.035~0.138)	0.567 (0.223~5.223)	y = 0.285 + 1.156x	17.11
Wheat populations-rice (WR)	0.003 (0.001~0.005)	0.022 (0.014~0.036)	y = 1.980 + 1.201x	4.89

x and y indicate the common logarithm of dosage and the probability value of the mortality conversion, respectively. Relative toxicity index was calculated against the LC_50_ value of the RR treatment (rice population reared on rice).

**Table 3:** Three-factor Scheirer–Ray–Hare for the effects of insecticide treatment and host switching treatment on the developmental duration of *C. medinalis* larvae at different instars.

Parameters	Source	df	Sum of Squares	*H*	*p*
Development duration of 3rd instar larvae	Insecticide treatment (A)	1	36,958.1618	10.2179	0.0014
	Population origin (B)	1	54,281.5429	15.0074	<0.001
	Host switching treatment (C)	1	33,897.1665	9.3716	0.0022
	A × B	1	3337.2848	0.9227	0.3368
	A × C	1	83,905.4122	23.1975	<0.001
	B × C	1	167,475.6088	46.3024	<0.001
	A × B × C	1	8733.7498	2.4146	0.1202
	Residuals	218	425,235.0731		
Development duration of 4th instar larvae	Insecticide treatment (A)	1	311,531.5885	73.7989	<0.001
	Population origin (B)	1	8203.2229	1.9433	0.1633
	Host switching treatment (C)	1	3151.9064	0.7467	0.3875
	A × B	1	79,593.4079	18.8549	<0.001
	A × C	1	27.2134	0.0064	0.9360
	B × C	1	5567.6333	1.3189	0.2508
	A × B × C	1	1055.7323	0.2501	0.6170
	Residuals	218	540,674.7952		
Development duration of 5th instar larvae	Insecticide treatment (A)	1	200,500.1607	47.9938	<0.001
	Population origin (B)	1	18,875.0213	4.5181	0.0335
	Host switching treatment (C)	1	9861.4806	2.3605	0.1244
	A × B	1	75,019.9308	17.9575	<0.001
	A × C	1	12,526.7073	2.9985	0.0833
	B × C	1	134,763.3334	32.2583	<0.001
	A × B × C	1	38,456.5723	9.2054	0.0024
	Residuals	218	449,963.2936		

**Table 4:** Scheirer–Ray–Hare test for the effects of insecticide treatment and host switching treatment on pupal weight, pupation rate, number of eggs laid per female, and egg hatching rate of *C. medinalis*.

Parameters	Source	df	Sum of Squares	*H*	*p*
Pupation rate	Insecticide treatment (A)	1	253,500	65.5835	<0.001
	Population origin (B)	1	12,255.1042	3.1705	0.07500
	Host switching treatment (C)	1	1585.137	0.4101	0.5219
	A × B	1	5367.1832	1.3886	0.2386
	A × C	1	53.0453	0.0137	0.9067
	B × C	1	50,851.7175	13.156	<0.001
	A × B × C	1	9731.9172	2.5178	0.1126
	Residuals	232	590,462.3956		
Pupal weight	Insecticide treatment (A)	1	53,633.1420	7.4731	0.0063
	Population origin (B)	1	495,846.3696	69.0899	<0.001
	Host switching treatment (C)	1	106.2632	0.0148	0.9032
	A × B	1	7.7768	0.0011	0.9737
	A × C	1	4586.2178	0.6390	0.4241
	B × C	1	51,780.1955	7.2149	0.0072
	A × B × C	1	14,769.6417	2.0580	0.1514
	Residuals	285	1,474,902.8940		
Number of eggs laid per female	Insecticide treatment (A)	1	948.2135	1.4920	0.2219
	Population origin (B)	1	491.4997	0.7733	0.3792
	Host switching treatment (C)	1	22.2989	0.0351	0.8514
	A × B	1	53.3675	0.0840	0.7720
	A × C	1	317.8147	0.5001	0.4795
	B × C	1	441.5946	0.6948	0.4045
	A × B × C	1	20.3424	0.0320	0.8580
	Residuals	79	52,361.8688		
Egg hatching rate	Insecticide treatment (A)	1	665.1545	1.0461	0.3064
	Population origin (B)	1	7934.9800	12.4799	<0.001
	Host switching treatment (C)	1	866.4231	1.3627	0.2431
	A × B	1	99.1281	0.1559	0.6930
	A × C	1	407.6157	0.6411	0.4233
	B × C	1	422.1950	0.6640	0.4151
	A × B × C	1	94.6075	0.1488	0.6997
	Residuals	79	44,190.3961		

**Table 5:** Two way ANOVA for the effects of insecticide treatment and host switching treatment on the expression levels of P450 and JH-related genes in *C. medinalis*.

Parameters	Source	df	Mean Squares	*F*	*p*
*CmedCYP49A1*	Insecticide treatment (A)	1	384.0000	7.6800	0.0056
	Population origin (B)	1	48.1667	0.9633	0.3263
	Host switching treatment (C)	1	486.0000	9.7200	0.0018
	A × B	1	0.1667	0.0033	0.9540
	A × C	1	6.0000	0.1200	0.7290
	B × C	1	48.1667	0.9633	0.3263
	A × B × C	1	48.1667	0.9633	0.3263
	Residuals	16	129.3333		
*CmedCYP6AE30*	Insecticide treatment (A)	1	32.6667	0.6533	0.4189
	Population origin (B)	1	24.0000	0.4800	0.4884
	Host switching treatment (C)	1	8.1667	0.1633	0.6861
	A × B	1	352.6667	7.0533	0.0079
	A × C	1	140.1667	2.8033	0.0941
	B × C	1	48.1667	0.9633	0.3263
	A × B × C	1	468.1667	9.3633	0.0022
	Residuals	16	76.0000		
*CmedCYP12A2*	Insecticide treatment (A)	1	468.1667	9.3633	0.0022
	Population origin (B)	1	504.1667	10.0833	0.0015
	Host switching treatment (C)	1	28.1667	0.5633	0.4529
	A × B	1	0.0001	0.0001	1.0000
	A × C	1	42.6667	0.8533	0.3556
	B × C	1	0.6667	0.0133	0.9081
	A × B × C	1	28.1667	0.5633	0.4529
	Residuals	16	78.0000		
*CmedCYP301A1*	Insecticide treatment (A)	1	322.6667	6.4533	0.0111
	Population origin (B)	1	80.6667	1.6133	0.2040
	Host switching treatment (C)	1	37.5000	0.7500	0.3865
	A × B	1	294.0000	5.8800	0.0153
	A × C	1	280.1667	5.6033	0.0179
	B × C	1	37.5000	0.7500	0.3865
	A × B × C	1	13.5000	0.2700	0.6033
	Residuals	16	84.0000		
*CmedCYP4AE76*	Insecticide treatment (A)	1	228.1667	4.5633	0.0327
	Population origin (B)	1	130.6667	2.6133	0.1060
	Host switching treatment (C)	1	240.6667	4.8133	0.0282
	A × B	1	308.1667	6.1633	0.0130
	A × C	1	37.5000	0.7500	0.3865
	B × C	1	112.6667	2.2533	0.1333
	A × B × C	1	13.5000	0.2700	0.6033
	Residuals	16	78.6667		
*CmedCYP4V2*	Insecticide treatment (A)	1	864.0000	17.2800	<0.001
	Population origin (B)	1	16.6667	0.3333	0.5637
	Host switching treatment (C)	1	60.1667	1.2033	0.2727
	A × B	1	112.6667	2.2533	0.1333
	A × C	1	20.1667	0.4033	0.5254
	B × C	1	1.5000	0.0300	0.8625
	A × B × C	1	4.1667	0.0833	0.7728
	Residuals	16	70.6667		
*CmedAT*	Insecticide treatment (A)	1	32.6667	0.6533	0.4189
	Population origin (B)	1	486.0000	9.7200	0.0018
	Host switching treatment (C)	1	216.0000	4.3200	0.0377
	A × B	1	150.0000	3.0000	0.0833
	A × C	1	96.0000	1.9200	0.1659
	B × C	1	54.0000	1.0800	0.2987
	A × B × C	1	80.6667	1.6133	0.2040
	Residuals	16	34.6667		
*CmedJHAMT*	Insecticide treatment (A)	1	192.6667	3.8533	0.0497
	Population origin (B)	1	486.0000	9.7200	0.0018
	Host switching treatment (C)	1	266.6667	5.3333	0.0209
	A × B	1	66.6667	1.3333	0.2482
	A × C	1	42.6667	0.8533	0.3556
	B × C	1	32.6667	0.6533	0.4189
	A × B × C	1	24.0000	0.4800	0.4884
	Residuals	16	38.6667		
*CmedJHE*	Insecticide treatment (A)	1	580.1667	11.6033	0.0007
	Population origin (B)	1	400.1667	8.0033	0.0047
	Host switching treatment (C)	1	0.6667	0.0133	0.9081
	A × B	1	0.0001	0.0001	1.0000
	A × C	1	8.1667	0.1633	0.6861
	B × C	1	4.1667	0.0833	0.7728
	A × B × C	1	2.6667	0.0533	0.8174
	Residuals	16	154.0000		
*CmedJHBP1*	Insecticide treatment (A)	1	450.6667	9.0251	0.0027
	Population origin (B)	1	486.0000	9.7327	0.0018
	Host switching treatment (C)	1	5.0417	0.1010	0.7507
	A × B	1	0.6667	0.0134	0.9080
	A × C	1	0.0417	0.0001	0.9770
	B × C	1	2.0417	0.0409	0.8398
	A × B × C	1	12.0417	0.2411	0.6234
	Residuals	16	192.0000		

## Data Availability

Data is contained within the article.

## Abbreviations

The following abbreviations are used in this manuscript:

ANOVA	Analysis of variance
AT	Allatotropin
CarE	Carboxylesterase
DIMBOA	2,4-dihydroxy-7-methoxy-1,4-benzoxazin-3-one
GSA	Genome Sequence Archive
GST	Glutathione S-transferase
JH	Juvenile hormone
JHAMT	Juvenile hormone acid methyltransferase
JHBP	Juvenile hormone binding protein
JHE	Juvenile hormone esterase
LC_25_	25% lethal concentration
LC_50_	50% lethal concentration
NGDC	National Genomics Data Center
P450	Cytochrome P450 monooxygenase
qPCR	Quantitative real-time PCR
RR	Rice-population reared on rice
RT-qPCR	Reverse transcription quantitative real-time PCR
RW	Rice-population reared on wheat
SE	Standard error
UGT	UDP-glycosyltransferase
WR	Wheat-population reared on rice
WW	Wheat-population reared on wheat
